# Vertical Infestation Profile of *Aedes* in Selected Urban High-Rise Residences in Malaysia

**DOI:** 10.3390/tropicalmed5030114

**Published:** 2020-07-07

**Authors:** Nurulhusna Ab Hamid, Siti Nurfadhlina Mohd Noor, Nur Rasyidah Isa, Rohaiyu Md Rodzay, Ainaa Mardia Bachtiar Effendi, Afiq Ahnaf Hafisool, Fatin Atirah Azman, Siti Farah Abdullah, Muhammad Khairi Kamarul Zaman, Mohd Iqbal Mohd Norsham, Noor Hasmiza Amanzuri, Nurliyana Abd Khalil, Izzah Farhah Zambari, Aimannur Najihah Mat Rani, Farah Diana Ariffin, Topek Omar, Nazni Wasi Ahmad, Han Lim Lee

**Affiliations:** 1 Medical Entomology Unit, WHO Collaborating Centre for Vectors, Institute for Medical Research, Ministry of Health, Malaysia, National Institutes of Health, Block C, Jalan Setia Murni U13/52, Seksyen U13, Setia Alam, Shah Alam 40170, Malaysia; snfadhlina@gmail.com (S.N.M.N.); nurrasyidahisa@gmail.com (N.R.I.); rohaiyuRodzay@gmail.com (R.M.R.); ainaaeffendi@gmail.com (A.M.B.E.); afiqahnaf94@gmail.com (A.A.H.); fatinazman94@gmail.com (F.A.A.); abdullahsitifarah@gmail.com (S.F.A.); eryian30@yahoo.com (M.K.K.Z.); iqbal_sham@live.com (M.I.M.N.); osakurataxo@yahoo.com (N.H.A.); liyana_khalil@yahoo.com (N.A.K.); izzahzambari@gmail.com (I.F.Z.); anmatrani@gmail.com (A.N.M.R.); farahdiana.a@moh.gov.my (F.D.A.); nazni@moh.gov.my (N.W.A.); mosquito7090@gmail.com (H.L.L.); 2 Entomology and Pest Unit, Federal Territory of Kuala Lumpur & Putrajaya Health Department, Jalan Cenderasari, Kuala Lumpur 50590, Malaysia; aks.topek@gmail.com

**Keywords:** vertical dispersal, *Aedes*, mosquito, high-rise residences, dengue, surveillance

## Abstract

Dengue is placing huge burdens on the Malaysian healthcare system as well as the economy. With the expansion in the number of high-rise residential buildings, particularly in the urban centers, the flight range and behavior of *Aedes* mosquitoes may be altered in this habitat type. In this study, we aimed to expand the understanding of the vertical distribution and dispersal of *Aedes* in nine selected high-rise residences in Kuala Lumpur, Selangor, and Johor using ovitraps as the sampling method. We discovered that *Ae. aegypti* is the predominant species in all study sites. Both *Ae. aegypti* and *Ae. albopictus* are most abundant within the first three levels and could be found up to level 21 (approximately 61.1–63.0 m). Pearson correlation analyses exhibited negative correlations in eight out of nine study sites between the ovitrap indexes (OIs) within each floor level, suggesting that *Aedes* density decreased as the building level increased. Our findings provide information to the public health authorities on ‘hot spot’ floors for effective suppression of dengue transmission.

## 1. Introduction

Dengue is a vector-borne infectious disease with an estimated 100–400 million infections annually [1]. Transmission of one of the four antigenically distinct serotypes of dengue virus (DENV-1 to DENV-4) may cause dengue and its severe forms—hemorrhagic fever and shock syndrome [2]. To date, efficacious and cost-effective vaccines and antiviral drugs against these four serotypes are still under various stages of research and development [3,4]. Until dengue vaccines or antiviral drugs become available, the only current method proven to be effective in dengue control and prevention is vector control measures that could be sustained through community involvement [1]. The spread of dengue globally is fueled by a combination of various factors including climate change, rapid urbanization, increased international travel and trade, as well as changes in land-use patterns [5].

Dengue is endemic in Malaysia and Malaysia is one of the most severely affected countries in South-East Asia [1]. The country had 80,615 reported dengue cases and 147 deaths in the year 2018 [6]. Selangor reported the highest number of dengue cases (45,349 cases), followed by Federal Territories of Kuala Lumpur and Putrajaya (7591 cases), Penang (6071 cases) and Johor (5885 cases) [6]. The primary mosquito vector for dengue is *Aedes aegypti*, while *Ae. albopictus* is recognized as the secondary vector [2]. These two *Aedes* species are prevalent in Malaysia where they can coexist in similar ecological niches [7].

Increased demand for housing due to urban sprawl and land scarcity in major urban regions resulted in an increase in high-rise residential buildings ranging from flats and apartments to luxury condominiums [8]. According to a housing statistic, 5.5 million people in Malaysia live in high-rise residences as of June 2014 [9], suggesting that a large number of Malaysians are experiencing high-rise living, also known as vertical living. The rise in the numbers of new non-landed, high-rise properties, as a result, may pose greater challenges in developing preventive and control measures against dengue, due to a change in dispersal dynamics of *Aedes* due to adaptation to non-landed, high-rise housing. 

The vector flight range plays a key role in the dynamics of transmission. In standard approaches by the Ministry of Health (MOH), Malaysia, reduction of the proliferation of infected vectors and suppression of dengue outbreaks are achieved through vector control measures, e.g., insecticide fogging, larviciding and elimination of potential larval habitats, within a buffer zone with a radius of 200 m from the house where a dengue case is reported [10]. This is in line with standard mark-release-recapture studies showing that the mean distance of recaptured *Ae. aegypti* rarely went beyond 100 m [11,12]. In a review article, *Ae. aegypti* is said to disperse horizontally with an average distance of 83.4 m [13]. However, the standard approaches used are generally applied to low-rise housing. Slightly different vector control approaches may be required for high-rise housing. At present, specific guidelines for vector control activities in high-rises are yet to be released. But according to a circular from the MOH, fogging shall be performed within a 200 m radius from the index case house, and for buildings that have more than five levels, fogging shall be completed at least within five levels below and above the index case house [14]. For buildings that have five floors or less, the whole building shall be fogged [14].

Studies on the vertical distribution of *Aedes* in high-rises have been carried out in several countries, including Sri Lanka [15], Trinidad [16], Singapore [17] and Malaysia [7,18,19]. Research in Trinidad, West Indies, suggested that *Aedes* eggs were found mostly in 13–24 m elevations [16]. While in Sri Lanka, *Aedes* could be found from the ground floor to the highest floor of 130 ft (approximately 40 m), but largely were discovered at the 60 ft elevation (approximately 18 m) [15]. A similar observation was seen in Putrajaya, Malaysia in which *Aedes* density peaked at the height of 16.1–18 m [19]. These studies indicated that infestations of *Aedes* in high-rises were extensive, ranging from the ground level to higher elevations, and that they have the ability to survive in high-rise buildings.

This study aimed to expand the understanding of the vertical distribution of *Aedes* in nine selected high-rise residences in the Federal Territory of Kuala Lumpur and the States of Selangor and Johor using ovitraps as the sampling method. Although a few studies have been performed in Malaysia for similar purposes [7,18,19,20], the ever-increasing number of high-rise residences in Malaysia makes this study warranted. Up-to-date information gained from this study will hopefully serve as guidelines for the public health officers to identify ‘hot spot’ floors of *Aedes* infestation in high-rises for quick and more efficient dengue control efforts.

## 2. Materials and Methods

### 2.1. Study Sites

**Figure 1:**
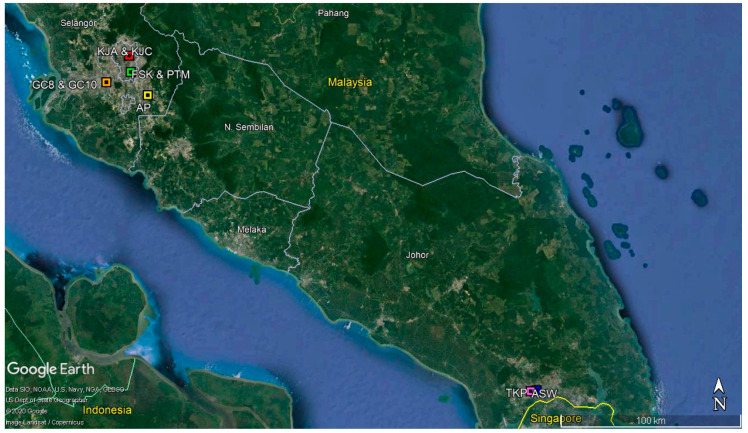
The geographical map of the study sites. Abbreviations: GC8—Goodyear Court 8, GC10—Goodyear Court 10, AP—Apartmen Pesona, FSK—Flat Sri Kota, PTM—PPR Taman Mulia, KJA—Kuarters Jalan Sultan Abdul Aziz Block A, KJC—Kuarters Jalan Sultan Abdul Aziz, TKP—PPR Taman Kempas Permai and ASW—Apartmen Sri Wangi.

The study was conducted in nine study sites that constituted high-rise properties. Five study sites were in Selangor State—Subang Perdana Goodyear Court 8 (GC8), Goodyear Court 10 (GC10), Apartmen Pesona (AP), Flat Sri Kota (FSK) and PPR Taman Mulia (PTM); two study sites were in Johor State—PPR Taman Kempas Permai (TKP) and Apartmen Sri Wangi (ASW) and two study sites were in the Federal Territory of Kuala Lumpur—Kuarters Jalan Sultan Abdul Aziz Block A (KJA) and Block C (KJC) (Figure 1). The sites were chosen because they had prolonged dengue outbreaks between the years 2014 and 2018. All study sites were in urban areas, at which their coordinates were obtained using the Global Positioning System (GPS) (Garmin Montana® 680, Olathe, Kansas, US). The physical and geographical description as well as the building design of each study site are explained in Supplementary Table S1 and Figures S1–S6.

### 2.2. Ovitrap Surveillance

The number of *Aedes* larvae that represented the population densities of *Aedes* was assessed at different building levels via ovitraps. Ovitrapping was performed as described in Lee [21]. Each ovitrap consisted of a 250 mL cylindrical, black plastic container (7.0 cm diameter, 9.0 cm height) filled with tap water to a level of 5.5 cm. Each ovitrap was equipped with a removable oviposition paddle that was made from a thin strip of brown hardboard (10 cm × 2.5 cm × 0.3 cm). The paddle was placed diagonally with the rough surface facing upwards where the mosquitoes laid eggs above the water level.

Ovitrap monitoring was conducted separately for each study site between the years 2014 and 2018, with sampling at each site consisting of 10 weeks of data collection. The total number of ovitraps placed in each study site is described in Table 1. One ovitrap was placed randomly on each level that had minimum human, physical and environmental disturbance. We placed the ovitraps in a semi-indoor environment, defined as the area outside of the house units but is still sheltered by the roof, e.g., shared corridor and stairway. The ovitraps were collected after seven days and replaced with new ovitraps consisting of fresh tap water and egg-free oviposition paddles. Any disturbances to the exposed ovitraps, e.g., theft, vandalism or invasion by insects, were recorded.

The collected ovitraps were transported back to the Institute for Medical Research (IMR), Kuala Lumpur, laboratory for further processing. The ovitrap contents along with the oviposition paddle were transferred into the plastic containers and labeled according to the study site, date of collection, and level. Beef liver powder (Difco Laboratories, MD, USA) was added into each container as larval food. Identification to species level of third or fourth instar was performed using established taxonomy keys [22,23] under a compound microscope (Nikon Eclipse^®^ E100, Japan). The ovitrap contents were examined for species identification until no newly emerged larvae were present in the containers.

### 2.3. Data Analysis 

Data entry and statistical analyses were conducted using Microsoft Excel and Statistical Package for Social Sciences (SPSS) Version 25.0 [24]. Ovitrap index (OI) is expressed as the percentage of positive ovitraps per the number of ovitraps recovered. Summation and the mean number of *Aedes* larvae for each *Aedes* species per ovitrap were performed in every study site. Paired samples *t*-test analysis was conducted to determine if there is any significant difference between the mean number of *Ae. aegypti* larvae and *Ae. albopictus* larvae per ovitrap. Single and cohabitation of *Aedes* spp. were also analyzed. Linear regression line for OIs in each floor level was constructed for each site. The Spearman’s rank correlation coefficient test was further conducted to investigate the correlation between the mean number of *Aedes* larvae and floor level. All levels of statistical significance were determined at *p* ≤ 0.05. 

## 3. Results

The overall results showed that the OI varied quite considerably between the nine study sites, with AP exhibiting the highest OI (63.00 ± 3.40%), followed by TKP (55.00 ± 2.40%) and ASW (51.00 ± 2.70%) (Table 2). The lowest OI belonged to KJA with OI of 26.00 ± 1.90% (Table 2). Paired samples *t*-test analysis indicated that the mean number of *Ae. aegypti* larvae per ovitrap was significantly higher than that of *Ae. albopictus* in all study sites (*p* ≤ 0.05), demonstrating that *Ae. aegypti* is the predominant species in all sites studied. Collected *Ae. aegypti* larval instars were highest in TKP (8847 larvae), while *Ae. albopictus* instars were most abundant in AP (980 larvae) (Table 2). The relative abundance (ratio) of *Ae. aegypti* to *Ae. albopictus* ranged from the lowest of 1.7:1 in AP to the highest of 30.6:1 in KJA (Table 2).

We performed a further analysis by looking at single and cohabitation of *Aedes* species in the same ovitraps. Our analyses showed that single breeding of *Aedes* was much more prevalent than the cohabitation of *Aedes* spp. in all study sites (Table 3). The proportions of ovitraps positive for single breeding of *Ae. aegypti* were noticeably high in all study sites, ranging from 64.80% in AP to 96.62% in KJA (Table 3). It is worth noting that KJA was the only study site void of cohabitation of *Aedes* spp., contributing to its high percentage of single breeding of *Ae. aegypti* (Table 3). Meanwhile, percentages of ovitraps positive for single breeding of *Ae. albopictus* were, in general, higher than cohabitation of *Aedes* spp., with the exception of TKP (Table 3). Analysis variance of one-way ANOVA showed that there were significant differences in the mean number of *Ae. aegypti* larvae (*p* ≤ 0.05, F = 34.652) and *Ae. albopictus* larvae (*p* ≤ 0.05, F = 9.712) in single breeding as well as in cohabitation of *Aedes* spp. among the nine study sites (*p* ≤ 0.05, F = 8.628). TKP displayed the highest mean number of *Ae. aegypti* per ovitrap in single breeding (17.33 ± 1.50) and cohabitation of *Aedes* spp. (3.58 ± 0.74) (Table 3). Interestingly, PTM was the only site that exhibited a higher ratio of *Ae. albopictus* to *Ae. aegypti* in cohabitation containers (Table 3).

**Figure 2:**
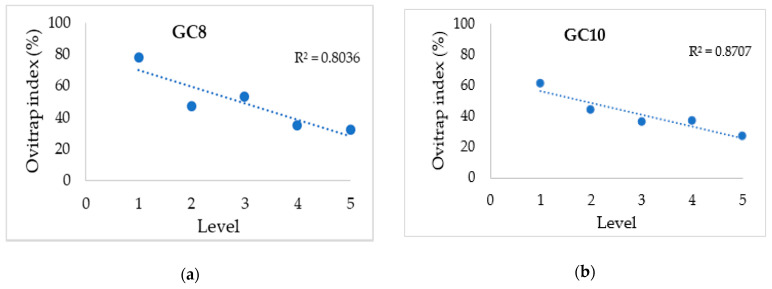

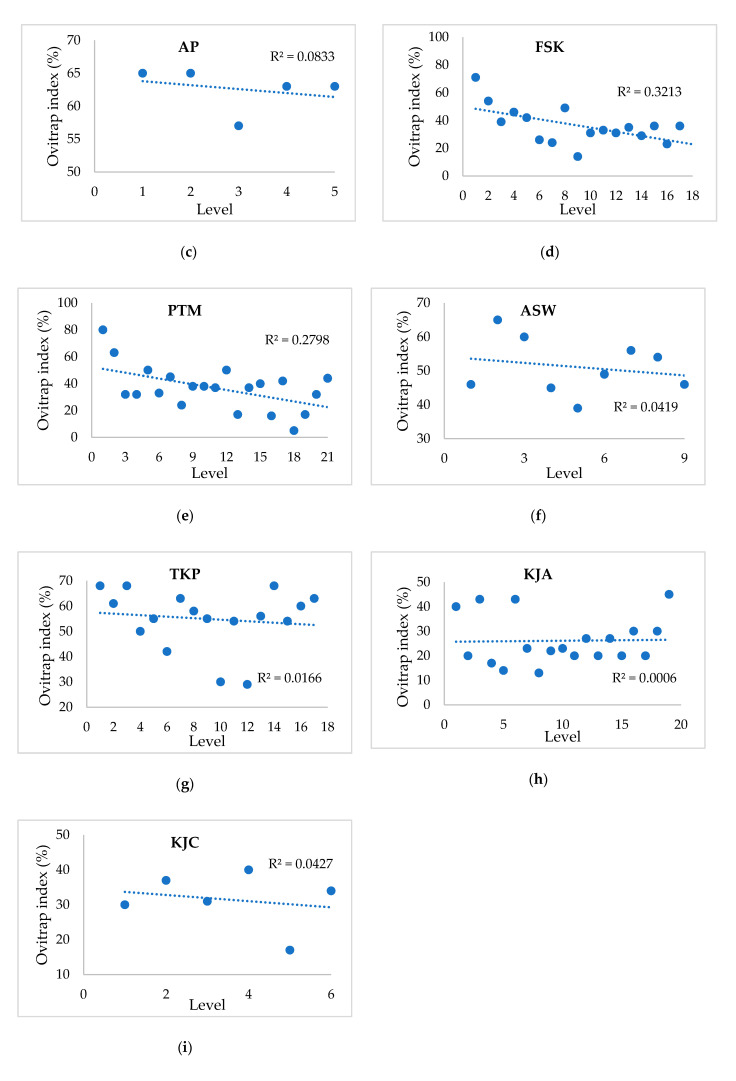
Relationship between ovitrap index (%) and level in each study site. (**a**–**i**) GC8, GC10, AP, FSK, PTM, ASW, TKP, KJA, KJC.

The relationship between OI and floor level was investigated in this study. For this, the linear regression line of each study site was constructed (Figure 2). Except for KJA, Pearson correlations exhibited negative correlations in all sites, suggesting that as the floor level increased, the OI decreased. Significant differences between OIs within each floor level were exhibited in GC8 (*p* = 0.020), GC10 (*p* = 0.010), FSK (*p* = 0.009) and PTM (*p* = 0.007). KJA had a positive correlation between OIs within each floor level but was insignificant *(p* = 0.460).

To go deeper into this analysis, we found that six out of nine study sites—GC8, GC10, AP, FSK, PTM, and TKP—displayed the highest mean number of *Aedes* larvae per ovitrap on floor level 1 (0.0–3.0 m height) (Table 4). In contrast, *Aedes* larvae per ovitrap peaked on the top floor (54.1–57.0 m height) in KJA (Table 4). In ASW and KJC, *Aedes* larvae density was highest on level 7 (18.1–21.0 m height) and level 4 (9.1–12 m height), respectively (Table 4). Intriguingly, all three sites of the five-story building—GC8, GC10 and AP—showed the lowest mean number of *Aedes* larvae per ovitrap on the highest floor (12.1–15.0 m height) (Table 4). Likewise, ASW, KJA and KJC presented the least *Aedes* larvae per ovitrap within the same floor height (Table 4). It may be noted that *Ae. aegypti* was found on every floor level at all study sites, up to level 21 (61.1–63.0 m height). Similar to *Ae. aegypti*, *Ae. albopictus* was discovered on each floor level up to level 21, but in KJA and KJC, the species could only be found up to level 3 and level 2, respectively (Supplementary Tables S2 and S3). Similar to the OI analysis, Spearman’s rank correlation coefficient showed negative correlations in all sites except for KJA (Table 4). 

## 4. Discussion

With the expansion in the number of high-rise residential buildings largely in the urban areas in Malaysia, we believe that the up-to-date information on the vertical distribution of *Aedes* in this habitat type is warranted. We discovered that *Ae. aegypti* was the predominant species in all nine study sites, in which the number of *Ae. aegypti* larval instars were up to 30-fold higher in comparison to *Ae. albopictus*. The dominance of *Ae. aegypti* is in line with the results of several vertical dispersal studies conducted in urban high-rise residences in Kuala Lumpur and Selangor [7,18] as well as Putrajaya [19]. This finding is not surprising, because *Ae. aegypti* is adapted to reside in and around human dwellings. Female *Ae. aegypti* also preferentially breed in artificial and domestic containers in the urban areas [25], which may be commonly found in the semi-indoor environment of our study sites. Likewise, the lower number of *Ae. albopictus* larval instars observed in all study sites were due to their behavior. *Ae. albopictus* prefers to breed in natural habitats such as tree holes and to reside where vegetation is plentiful [26], which may be especially uncommon in high-rise properties where space is limited for planting or gardening. However, recent studies in Malaysia discovered that *Ae. albopictus* have been acclimatizing to the domestic environment in urban areas. The species is now found both in the indoor and outdoor environments and readily breeds in artificial or man-made containers [27,28]. Therefore, it is not surprising that *Ae. albopictus* was found, but in a lower number.

The mixed infestation of *Ae. aegypti* and *Ae. albopictus* was detected in all sites, apart from KJA, accounting for 1.39% to 13.60% of the percentage of positive ovitraps collected. The cohabitation of *Aedes* spp. found in the present study suggested that their ecological niche overlapped. A study in multi-story buildings in Selangor and Kuala Lumpur by Lau [7] discovered a higher percentage of cohabitation of *Aedes* spp., ranging from 10.77% to 26.56% from the total percentage of positive ovitraps collected. The lower percentages of co-breeding compared to single breeding may be explained through their breeding behavior. *Ae. albopictus*, for instance, would avoid breeding in habitats occupied by *Ae. aegypti*, and vice versa, as shown in a study in Penang [29].

The discovery of *Aedes* on each level suggested that *Aedes* could disperse up to the highest floor level. Three main types of adult mosquito dispersal exist [30]. The first is unintentional dispersal by riding along with humans via airplanes or ships, involving long-distance dispersal from one continent to another. The second is mosquitoes being carried through wind-assisted dispersal, also in a long-distance and unintentional dispersal. The third type (involved in our study) is considered as active and intentional in a short-distance dispersal. In high-rise buildings, *Aedes* could be involved in short-distance dispersal via human transportation using stairs or lifts as suggested in other studies [7,19]. Mosquitoes may disperse to look for blood sources, nectar sources, mates, oviposition sites and resting sites [30]. The search for new oviposition sites, for example, could be represented by the ovitraps and possibly other cryptic breeding sites that drive the dispersal of female *Aedes* through the availability of stagnant water for the development of immature, aquatic stages of mosquitoes. Studies conducted in urban habitats in Malaysia discovered that both *Aedes* spp. were mostly found in domestic receptacles such as flower vases, flower pot plates, pails, bowls, refrigerator trays, plastic containers, empty paint cans and in building structures such as roofs, drains, gutters and gully traps [27,31,32]. We hypothesized that the *Aedes* mosquitoes in our study sites could have originated from these sources.

Additionally, the discovery of *Aedes* on each level suggested that the ground floor, as well as the higher elevations, support the survival of *Aedes*. The availability of biotic and abiotic components (ecological niche) and ecosystem in our study sites may have provided sufficient bloodmeal, resting place, and breeding sites for *Aedes* [16]. Biotic components include humans, pets, and plants, while abiotic components include temperature, humidity, wind speed, and building structure [16]. The denser people living in high-rises will most likely provide more blood meals for *Aedes*, and in turn increase their vectorial capacity to transmit dengue viruses due to higher contact with human hosts [33]. This is particularly true for *Ae. aegypti*, which has an anthropophilic tendency for preferential feeding on human blood and takes multiple blood-feeding in a gonotrophic cycle [34,35]. 

Although *Aedes* dispersal seemed extensive (up to level 21, 61.0–63.0 m height), the present study demonstrated that *Aedes* larvae were most abundant on the ground floor. A plausible reason may be due to the existence of communal areas in the ground level such as parking spaces, shops and waste disposal areas that sometimes could have poor sanitation, creating favorable mosquito breeding sites. In Singapore, for example, children residing in the ground level reportedly had higher dengue infection rates compared to those residing in higher levels because more *Aedes* breeding habitats were found in the communal areas [17]. This finding emphasized that the ground floor demands additional efforts during vector control measures. 

Intriguingly, unlike the other eight other study sites, we found *Aedes* larvae were most abundant at the highest level of KJA with an elevation of approximately 54.1 to 57 m. We speculate that this might be due to the presence of an uneven rooftop floor, where heavy rain will cause puddles and create breeding sites for mosquitoes. Importantly, in the study, we found that the maximum height of *Ae. aegypti* dispersal was identical to *Ae. albopictus* (61.1–63.0 m). However, we wanted to point out that *Ae. albopictus* had overall a higher density closer to the ground floor. In KJA and KJC, they could only be found up to level 2 and level 3, respectively. The results suggested that *Ae. albopictus* preferred elevations closer to the ground floor, presumably with scattered vegetation around for breeding and resting. Furthermore, from the results, it was clear that ‘hot spot’ floors of *Aedes* infestation were within the first three floors. Again, we think that floors closer to the ground may create a complete ecosystem and ecological niche suitable for *Aedes* infestation.

It is noteworthy to point out that TKP exhibited comparably high *Aedes* populations at the upper levels (levels 14 to 17) as well as the first two floor levels, implying a U-shaped curve instead of a declining trend. This may have occurred owing to the existence of water tanks between levels 16 and 17. Likewise, PTM has water tanks between levels 20 and 21 that may be responsible for the considerably large *Aedes* populations at the upper levels.

Our study possesses some limitations. Firstly, we did not consider environmental factors such as temperature, relative humidity, wind speed and rainfall. Wind speed, for instance, could influence the flight capabilities of mosquitoes [36], while relative humidity influences the mating and feeding behavior of mosquitoes [37]. Future research incorporating these environmental factors may be warranted. Secondly, our study sites have different building designs that may have influenced our results. However, we could say that the low-cost houses, i.e., AP, FSK, TKP, and PTM, possess similar building designs (Supplementary Figures S2, S3 and S5). Because our study only included low- and medium-cost houses, future studies incorporating high-cost houses such as luxury condominiums may provide interesting new insights into the vertical dispersal of *Aedes*.

In conclusion, our study revealed that the dispersal of *Aedes* vertically in high-rise residential buildings could be considered extensive. We think that *Aedes* could disperse to the upper floors even under little pressure to fulfill their needs, particularly finding hosts and oviposition sites. Based on our results, we recommended that the vector control operations should be concentrated within the first three floors and that guidelines specific for high-rises should be available. Given the extensive vertical dispersal of *Aedes*, other levels (middle and upper) should also be taken into consideration during vector control measures, depending on the location of the index case house as recommended in the circular. Vertical vector dispersal and propagation may play key roles in dengue virus dissemination in high-rises and may require further attention to fully investigate its importance.

## Figures and Tables

**Table 1:** The total number of ovitraps placed in each study site.

Study Site	Total Number of Ovitraps
GC8	300
GC10	500
AP	200
FSK	700
PTM	400
ASW	360
TKP	510
KJA	570
KJC	190

**Table 2:** The overall analysis of *Aedes* larval instars collected in each study site.

Study Site	Ovitrap Collected	Ovitrap Index (%)	*Ae. aegypti*		*Ae. albopictus*		Ratio *Ae. aegypti*: *Ae. albopictus*
			Total Larvae	Overall Percentage (%)	Total Larvae	Overall Percentage (%)	
GC8	297/300	48.00 ± 2.90	5216	94.02	332	5.98	15.7: 1
GC10	454/500	41.00 ± 2.30	5258	84.63	955	15.37	5.5: 1
AP	200/200	63.00 ± 3.40	1640	62.60	980	37.40	1.7: 1
FSK	658/700	37.00 ± 1.90	3111	91.10	304	8.90	10.3: 1
PTM	371/400	37.00 ± 2.50	1614	76.17	505	23.83	3.2: 1
ASW	351/360	51.00 ± 2.70	4483	90.22	486	9.78	9.3: 1
TKP	448/510	55.00 ± 2.40	8847	92.45	722	7.55	12.3: 1
KJA	564/570	26.00 ± 1.90	1898	96.79	63	3.21	30.6: 1
KJC	188/190	31.00 ± 3.40	738	94.98	39	5.02	18.7: 1

**Table 3:** Comparisons between single breeding and cohabitation of *Aedes* spp. in each study site.

Study Site	Single Breeding				Cohabitation		
	*Ae. aegypti*		*Ae. albopictus*				
	Percentage of Positive Ovitraps (%)	Mean Larvae per Ovitrap ± SE	Percentage of Positive Ovitraps (%)	Mean Larvae per Ovitrap ± SE	Percentage of Positive Ovitraps (%)	Mean Larvae per Ovitrap ± SE	Ratio *Ae. aegypti*: *Ae. albopictus*
GC8	90.97	16.96 ± 1.66	5.56	0.79 ± 0.37	3.47	0.93 ± 0.45	1.86: 1.00
GC10	78.38	10.56 ± 0.99	13.51	1.40 ± 0.39	8.11	1.73 ± 0.57	1.45: 1.00
AP	64.80	7.38 ± 0.91	21.60	4.14 ± 1.18	13.60	1.59 ± 0.45	1.08: 1.00
FSK	90.24	4.55 ± 0.34	6.91	0.38 ± 0.11	2.85	0.26 ± 0.10	2.31: 1.00
PTM	78.68	4.25 ± 0.50	18.38	1.16 ± 0.31	2.94	0.30 ± 0.16	1.00: 1.90
ASW	79.33	11.32 ± 1.22	10.61	0.89 ± 0.27	10.61	1.95 ± 0.51	2.10: 1.00
TKP	82.26	17.33 ± 1.50	6.05	0.45 ± 0.16	11.69	3.58 ± 0.74	2.92: 1.00
KJA	96.62	3.37 ± 0.30	3.38	0.11 ± 0.07	0.00	0.00 ± 0.00	0.00: 0.00
KJC	94.92	3.76 ± 0.53	3.39	0.11 ± 0.09	1.69	0.27 ± 0.27	1.78: 1.00

**Table 4:** The mean number of *Aedes* larvae per ovitrap on each level. Spearman’s rank correlation coefficient between the mean number of *Aedes* larvae and level (height) was calculated using SPSS statistics.

Level	Approximate Height (m)	GC8	GC10	AP	FSK	PTM	ASW	TKP	KJA	KJC
1	0.0–3.0	36.16 ± 5.02	26.72 ± 3.71	14.90 ± 2.80	9.88 ± 1.40	14.10 ± 3.40	11.21 ± 3.08	36.29 ± 8.81	5.13 ± 1.73	4.10 ± 1.48
2	3.1–6.0	19.28 ± 3.91	12.99 ± 2.07	13.28 ± 2.68	6.57 ± 1.66	9.16 ± 2.62	15.83 ± 3.68	33.39 ± 8.59	1.67 ± 0.77	4.93 ± 1.53
3	6.1–9.0	14.22 ± 2.76	10.73 ± 2.25	14.18 ± 3.68	3.63 ± 1.05	6.05 ± 2.27	17.38 ± 3.82	20.21 ± 4.00	3.73 ± 1.07	3.76 ± 1.20
4	9.1–12.0	13.72 ± 3.34	10.04 ± 1.97	12.08 ± 3.99	6.23 ± 1.60	7.58 ± 3.56	9.38 ± 2.68	23.42 ± 7.21	2.59 ± 1.13	5.47 ± 1.52
5	12.1–15.0	10.53 ± 2.73	8.76 ± 2.08	11.08 ± 2.19	5.87 ± 1.42	7.83 ± 2.90	7.89 ± 2.67	17.90 ± 4.86	1.31 ± 0.91	2.23 ± 1.11
6	15.1–18.0				4.64 ± 1.59	5.44 ± 3.49	15.64 ± 4.04	14.75 ± 5.71	4.67 ± 1.12	4.31 ± 1.71
7	18.1–21.0				4.92 ± 1.81	5.45 ± 2.31	19.79 ± 5.25	25.25 ± 7.09	4.30 ± 1.75	
8	21.1–24.0				8.38 ± 1.81	3.12 ± 1.79	15.64 ± 4.41	17.21 ± 5.65	2.27 ± 1.28	
9	24.1–27.0				2.76 ± 1.51	5.50 ± 2.13	14.51 ± 4.21	12.86 ± 4.38	2.22 ± 0.97	
10	27.1–30.0				4.08 ± 1.28	3.25 ± 1.93		16.55 ± 8.33	4.07 ± 1.59	
11	30.1–33.0				1.53 ± 1.48	8.11 ± 3.44		19.61 ± 7.04	2.73 ± 1.09	
12	33.1–36.0				3.72 ± 1.29	9.06 ± 2.94		5.54 ± 2.86	3.03 ± 1.01	
13	36.1–39.0				3.15 ± 1.03	1.28 ± 0.80		23.00 ± 6.57	4.00 ± 1.68	
14	39.1–42.0				4.00 ± 1.41	4.58 ± 1.45		33.68 ± 7.43	2.80 ± 1.01	
15	42.1–45.0				5.23 ± 1.49	5.10 ± 2.69		10.86 ± 2.93	2.90 ± 1.23	
16	45.1–48.0				3.08 ± 1.10	0.89 ± 0.57		32.48 ± 8.58	2.97 ± 1.09	
17	48.1–51.0				5.31 ± 1.71	6.68 ± 2.46		17.73 ± 5.33	3.07 ± 1.18	
18	51.1–54.0					0.05 ± 0.05			3.90 ± 1.33	
19	54.1–57.0					1.17 ± 0.76			8.66 ± 2.01	
20	57.1–60.0					6.00 ± 2.29				
21	60.1–63.0					7.28 ± 4.52				
r		−0.304	−0.231	−0.070	−0.172	−0.203	−0.024	−0.059	0.024	−0.028
*P*		0.000	0.000	0.322	0.000	0.000	0.657	0.211	0.576	0.705

## Supplementary Materials

The following are available online at https://www.mdpi.com/2414-6366/5/3/114/s1, Table S1: Physical and geographical descriptions of all study sites. Figure S1–S6: Building designs of all study sites. Table S2: Total number and percentage of *Aedes* spp. on each level at KJA. Table S3: Total number and percentage of *Aedes* spp. on each level at KJC.
