# Supplementary material for: Vertical Infestation Profile of Aedes in Selected Urban High-Rise Residences in Malaysia
Source: Trop Med Infect Dis. 2020 Jul 7;5(3):114. doi: 10.3390/tropicalmed5030114 (PMC7557596; doi:10.3390/tropicalmed5030114)
Supplement: Supplementary file 1 [file tropicalmed-05-00114-s001.pdf]

Supplementary

Table S1. Description of the study sites.

Study site	Geographical description	Physical description
Subang Perdana Goodyear Court 8 (GC8)	- 3°02'21.1"N101°35'32.8"E<br>- located in Subang Jaya, Selangor	- medium-cost houses<br>- consists of 6 blocks, 5 floors<br>- 8 units/ floor
Subang Perdana Goodyear Court 10 (GC10)	- 3°02'10.7"N 101°35'32.5"E<br>- located in Subang Jaya, Selangor	- medium-cost houses<br>- consists of 10 blocks, 5 floors<br>- 8 units/ floor
Apartmen Pesona (AP)	- 2°58’26.0"N 101°47’58.1"E<br>- located in Kajang, Selangor	- low-cost houses<br>- consists of 5 blocks, 5 floors<br>- 10 units/floor
Flat Sri Kota (FSK)	- 3°05'21.0"N 101°42'50.8"E<br>- located in Cheras, Selangor	- low-cost houses<br>- consists of 3 blocks, 17 floors<br>- 20 units/ floor
PPR Taman Mulia (PTM)	- 3°05'24.5"N 101°42'47.8"E<br>- located in Cheras, Selangor	- low-cost houses<br>- consists of 3 blocks, 21 floors<br>- 20 units/ floor
Apartmen Sri Wangi (ASW)	- 1°30'37.3"N 103°40'58.0"E<br>- located in Johor Bahru, Johor	- medium-cost houses<br>- consists of 4 blocks, 9 floors<br>- 8 units/ floor
PPR Taman Kempas Permai (TKP)	- 1°30’49.0"N 103°42'9.8"E<br>- located in Johor Bahru, Johor	- low-cost houses<br>- consists of 3 blocks, 17 floors<br>- 20 units/ floor
Kuarters Jalan Sultan Abdul Aziz, Block A (KJA)	- 3°10'11.5"N 101°42'19.7"E<br>- located in Kuala Lumpur	- staff quarters<br>- consists of 1 block, 19 floors<br>- 10 units/ floor
Kuarters Jalan Sultan Abdul Aziz, Block C (KJC)	- 3°10'09.2"N 101°42'23.1"E<br>- located in Kuala Lumpur	- staff quarters<br>- consists of 1 block, 6 floors<br>- 8 units/ floor

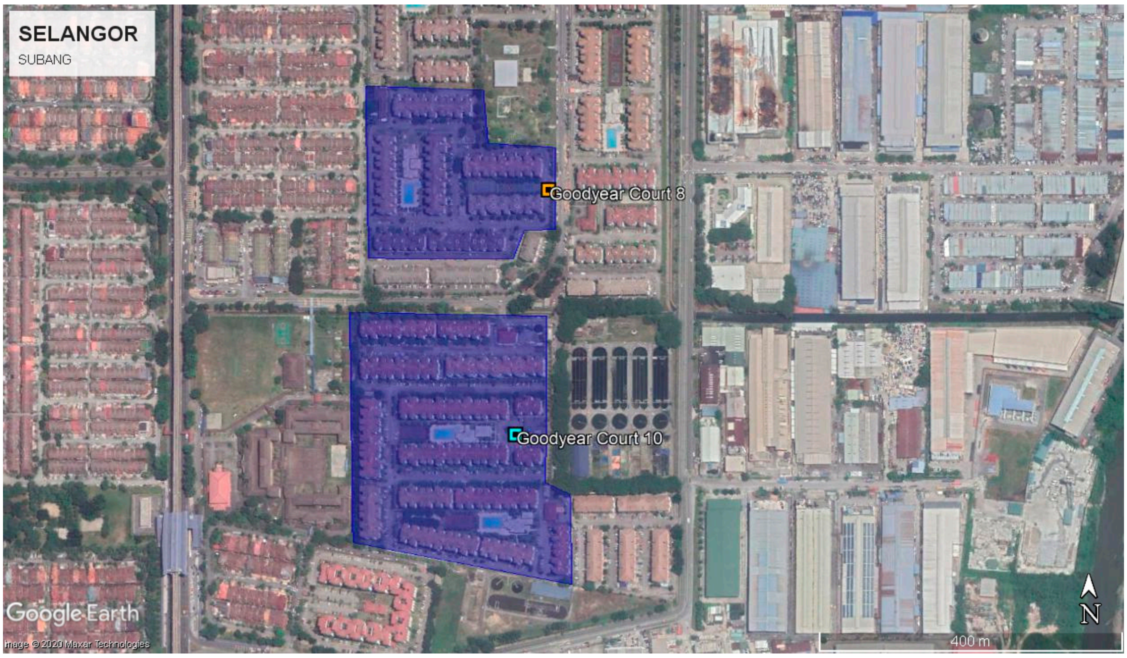

Figure S1: The building design of Subang Perdana Goodyear Court 8 and Goodyear Court 10.

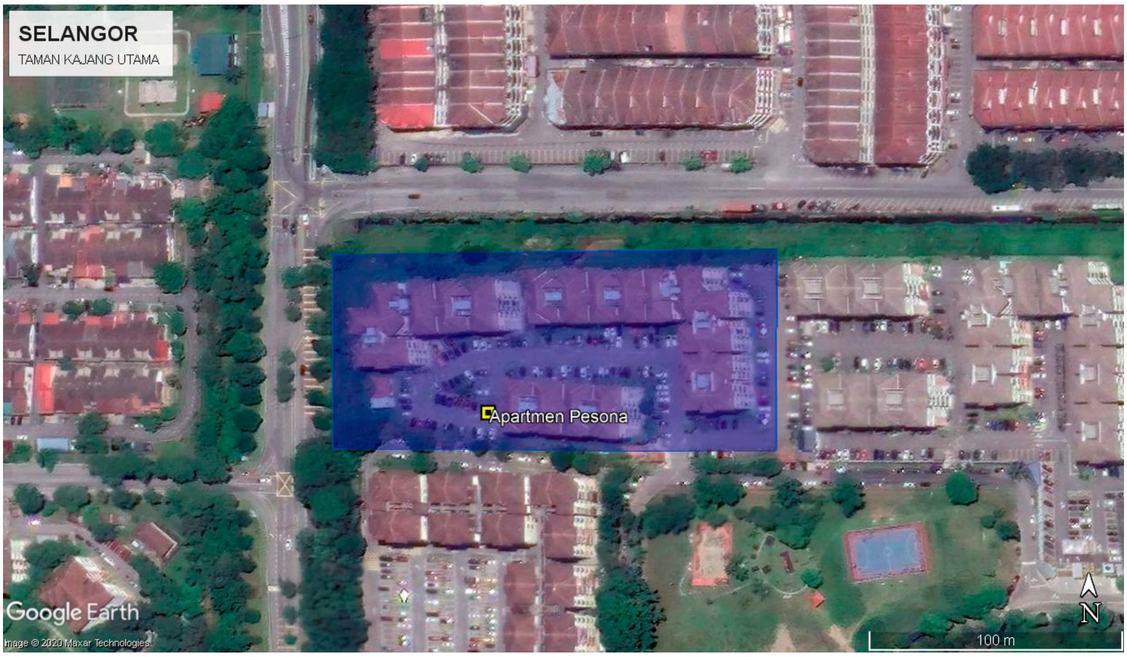

Figure S2: The building design of Apartmen Pesona.

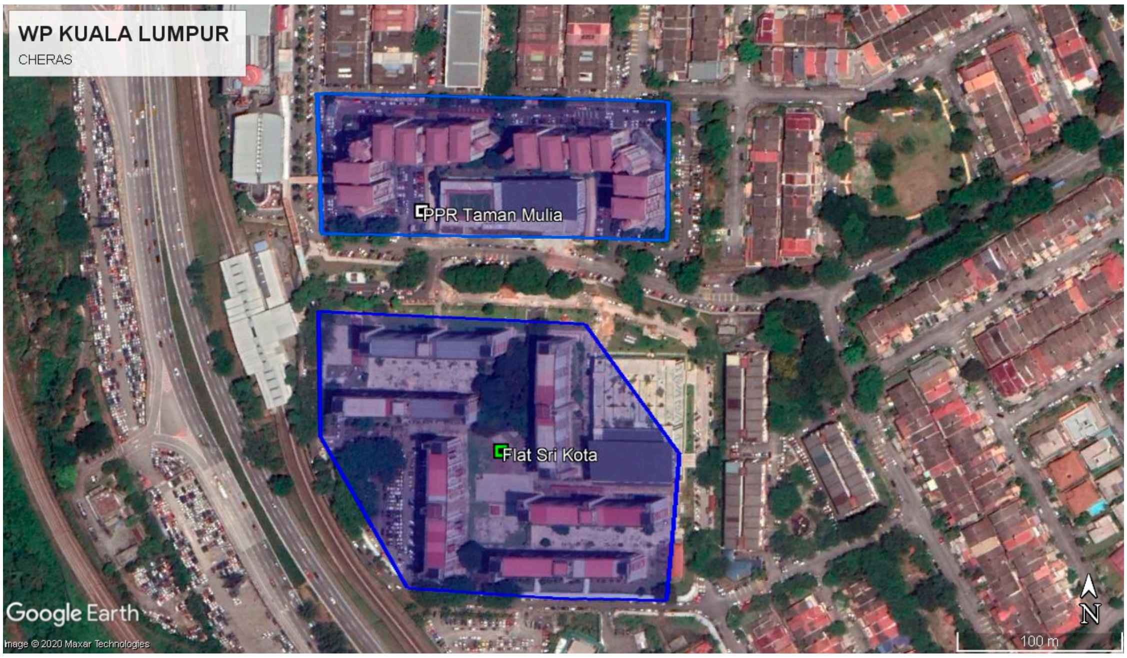

Figure S3: The building design of Flat Sri Kota and PPR Taman Mulia.

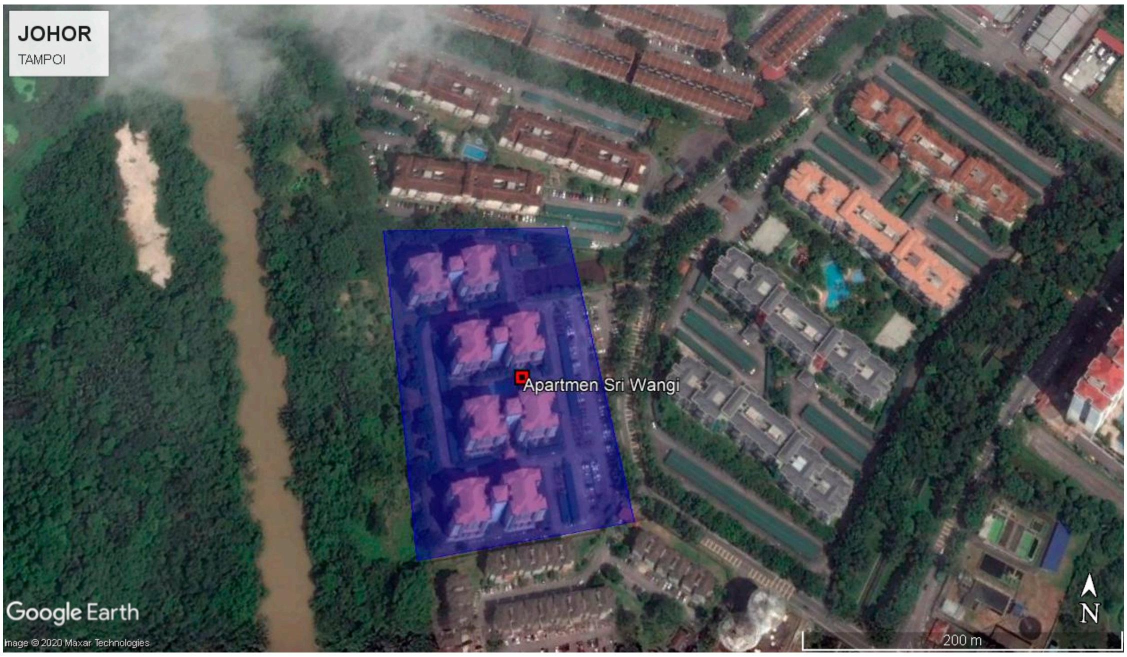

Figure S4: The building design of Apartmen Sri Wangi.

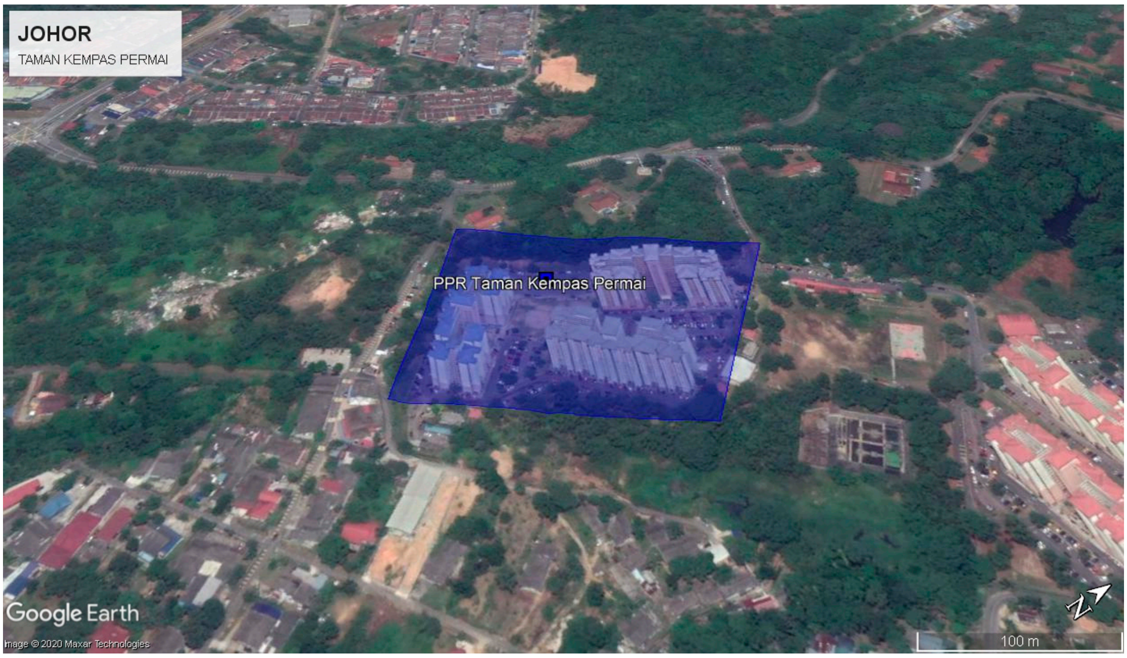

Figure S5: The building design of PPR Taman Kempas Permai.

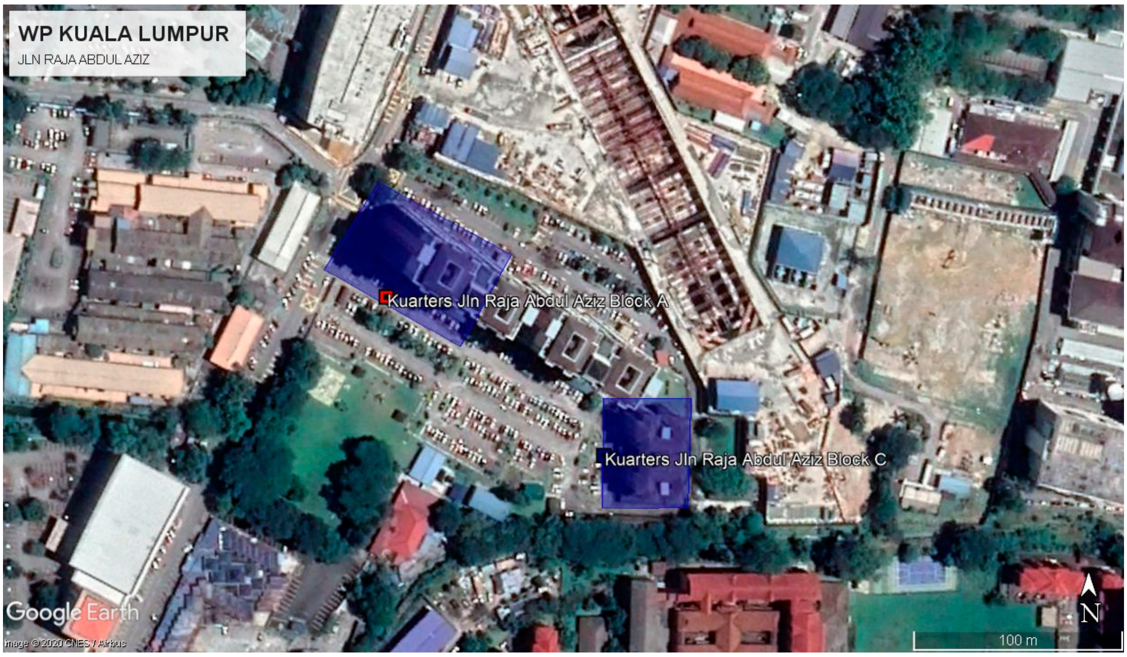

Figure S6: The building design of Kuarters Jalan Raja Abdul Aziz of Block A and Block C.

Table S2: Total number and percentage of *Aedes* spp. on each level at Kuarters Jalan Raja Abdul Aziz (Block A).

Level	Approximate height (m)	*Ae. aegypti*		*Aedes albopictus*	
		Total larvae	%	Total larvae	%
1	0.00 – 3.10	103	66.88	51	33.12
2	3.11 – 6.20	50	100.00	0	0.00
3	6.21 – 9.30	100	89.29	12	10.71
4	9.31 – 12.40	75	100.00	0	0.00
5	12.41 – 15.50	38	100.00	0	0.00
6	15.51 – 18.60	140	100.00	0	0.00
7	18.61 – 21.70	129	100.00	0	0.00
8	21.71 – 24.80	68	100.00	0	0.00
9	24.81 – 28.90	60	100.00	0	0.00
10	28.91 – 31.00	122	100.00	0	0.00
11	31.01 – 34.10	82	100.00	0	0.00
12	34.11 – 37.20	91	100.00	0	0.00
13	37.21 – 40.30	120	100.00	0	0.00
14	40.31 – 43.40	84	100.00	0	0.00
15	43.41 – 46.50	87	100.00	0	0.00
16	46.51 – 49.60	89	100.00	0	0.00
17	49.61 – 52.70	92	100.00	0	0.00
18	52.71 – 55.80	117	100.00	0	0.00
19	55.81 – 58.90	251	100.00	0	0.00

Table S3: Total number and percentage of *Aedes* spp. on each level at Kuarters Jalan Raja Abdul Aziz (Block C).

Level	Approximate height (m)	*Ae. aegypti*		*Ae. albopictus*	
		Total larvae	%	Total larvae	%
1	0.00 – 3.00	142	86.59	22	13.41
2	3.01 – 6.00	131	88.51	17	11.49
3	6.01 – 9.00	109	100.00	0	0.00
4	9.01 – 12.00	164	100.00	0	0.00
5	12.01 – 15.00	67	100.00	0	0.00
6	15.01 – 18.00	125	100.00	0	0.00
